# The role of assessment in supporting the movement toward patient-centred care

**DOI:** 10.1007/s40037-016-0318-9

**Published:** 2016-12-16

**Authors:** John J. Norcini

**Affiliations:** Foundation for Advancement and International Medical Education and Research, Philadelphia, USA

The Institute of Medicine describes patient-centredness as ‘Providing care that is respectful of and responsive to individual patient preferences, needs, and values, and ensuring that patient values guide all clinical decisions [1].’ The World Health Organization expands on this concept by describing people-centred care as being responsive to communities as well as patients [2]. Regardless of the definition, there is evidence that these forms of delivery improve both patient outcomes and the efficiency of care [3].

Unfortunately, the important study by Wilcox and colleagues in this edition of the journal finds that student assessments of the patient-centredness of the learning environment decrease as they progress from the first to second half of medical school [4]. The authors correctly conclude that this highlights the need to explicitly teach these skills throughout medical school with particular attention to the behaviours modelled by faculty during clinical training. These reforms will certainly be an important step forward in underscoring the importance of patient-centred care and enabling its implementation in practice.

In addition to these curricular recommendations, it would also be useful to focus on the role of assessment in the transformation of the curriculum to one that supports patient-centred care. Assessment plays a central role in both education and regulation across the entire continuum of training and practice. It enables change by signalling which competencies are important; it enhances and creates competence when it is used *for learning*; and it ensures competence when it is used as a measure *of learning*.

## Enable change

It is widely agreed that assessment has a significant educational effect [5]. It signals what is valued and it motivates trainees to prepare in a fashion that has educational benefit. Given these effects, it can enable the transition to patient-centred care by indicating the needed competencies and ensuring that they will be taught and modelled throughout the educational process. Just as importantly, if these competencies are not assessed, it will undermine change even if the competencies are given priority in the curriculum.

Interprofessional learning and collaborative practice is an example of a competence necessary for patient-centred care. It has been shown to generate positive health outcomes and increase patient safety while reducing complications and length of stay [6]. If this competence was included in the curriculum, students would be given the opportunity to acquire it. If it was assessed as well, it would signal the value of interprofessional learning and collaboration, increase students’ motivation to develop skills in the area, and enable, though not create, change in clinical practice. Although imperfect in a variety of ways, methods to assess this competency are already available and include written tests of roles and attitudes, simulation of teams, and peer and patient surveys.

## Enhance and create learning

Formative assessment (*for learning*) can have powerful effects in education and appropriately done it should focus on the needs of the student. It guides and creates learning while increasing the efficiency of the educational process. Reviews of the literature have highlighted the fact that feedback has a significant effect on learning and, although it is not often acknowledged, assessment is the predicate to feedback [7]. Therefore, formative assessment aimed at aspects of patient-centred care should be included as part of any curricular reform. Further, this will not require additional resources since the current overemphasis on summative assessment in many medical schools can be reduced substantially to provide the means for such a change.

Even when feedback is not provided, assessment by itself has a positive effect on learning. In an ingenious series of studies, Roediger and colleagues demonstrated that the active retrieval of information or performances required by assessment generates learning [8]. This constitutes another argument for increasing the number of formative assessments aimed at patient-centred care.

## Ensure competence

Summative assessment (*of learning*) is also a powerful, if overused, force in education and appropriately done it focuses on the needs of various stakeholders (such as patients, the health care system, and educational institutions) rather than students. Inclusion of the knowledge and skills required for patient-centred care in the summative examinations given during training will ensure that they become an integral part of the curriculum, that their value is communicated, and that students achieve competence.

In addition to their use in medical school, national high stakes assessment programmes are central to ensuring competence and there is a growing literature indicating that performance on such exams is associated with the outcomes of care. For example, studies have demonstrated a decrease in relative risk for mortality among the patients of those doctors who performed well on national assessment programmes [9, 10]. Moreover, inclusion of this content in national examinations will ensure that all medical schools in the country include content relevant to patient-centred care in their curricula. Most importantly, this will reassure patients that their physicians are proficient in this important competence.

In summary, the study by Wilcox et al. in this edition of the journal finds that the patient-centredness of the learning environment decreases as students move through their undergraduate educational experiences [4]. They underscore the need to explicitly teach these skills with particular attention to the behaviours modelled by faculty during clinical training. In this commentary, I have suggested that the reform they recommend also depends on changing the assessments done during and after medical school. Assessment plays a pivotal role in creating change across the continuum of training and practice. It signals what is valued. When used for formative purposes, it supports and creates learning for students and when used for summative purposes it ensures patients that their doctors have the required competencies. If assessment is not changed at the same time as the curriculum is changed, reform is doomed to failure.
